# Landscape of NcRNAs involved in drug resistance of breast cancer

**DOI:** 10.1007/s12094-023-03189-3

**Published:** 2023-04-17

**Authors:** Yujuan Kang

**Affiliations:** Department of Breast Surgery, The Affiliated Yantai Yuhuangding Hospital of Qingdao University, Yantai, China

**Keywords:** Breast cancer, Resistance, NcRNAs, Chemotherapy, Endocrine therapy, Targeted therapy, ASOs

## Abstract

Breast cancer (BC) leads to the most amounts of deaths among women. Chemo-, endocrine-, and targeted therapies are the mainstay drug treatments for BC in the clinic. However, drug resistance is a major obstacle for BC patients, and it leads to poor prognosis. Accumulating evidences suggested that noncoding RNAs (ncRNAs) are intricately linked to a wide range of pathological processes, including drug resistance. Till date, the correlation between drug resistance and ncRNAs is not completely understood in BC. Herein, we comprehensively summarized a dysregulated ncRNAs landscape that promotes or inhibits drug resistance in chemo-, endocrine-, and targeted BC therapies. Our review will pave way for the effective management of drug resistance by targeting oncogenic ncRNAs, which, in turn will promote drug sensitivity of BC in the future.

## Introduction

Breast cancer (BC) is a significant global health challenge [1]. It is a heterogeneous disease, involving numerous categories. There are five main categories of BC, stratified by the expressions of estrogen receptor (ER), progesterone receptor (PR), human epidermal growth factor receptor 2 (HER2), and Ki-67. The corresponding categories are Luminal A (LA), Luminal B (LB), Human epidermal growth factor receptor 2 (HER2) + , Normal breast-like (NBl) and Basal subtype [Triple negative breast cancer (TNBC)] [2, 3]. LA tumors typically show strong ER and PR levels and scarce HER2 and Ki-67 levels. LB cancers display strong ER and PR levels, strong or weak HER2 levels, and elevated Ki-67 levels. Given their distinct gene expressions, LA and LB tumors are generally more responsive to endocrine therapy, compared to chemotherapy [4]. In contrast, HER2 tumors have no ER and PR expressions, instead, they express HER2 and Ki-67. HER2 tumors are, therefore, better managed with targeted therapies, and adequately respond to neoadjuvant chemotherapy [4, 5]. The NBl form expresses ER and PR, and does not express HER2 and Ki-67. Therefore, these also respond well to chemotherapy. Lastly, TNBC responds well to neoadjuvant chemotherapy, however, the distant recurrence rates are markedly higher than other cancer forms [4]. Despite massive developments in various treatment regimen, a large quantity of patients still experienced disease recurrence and reduced survival due to new or acquired resistance to treatments, which, in turn, enhances metastatic risk [6]. Unfortunately, once metastasis occurs, the five-year overall survival (OS) rate becomes less than 25% [7]. Numerous cancer drug resistance pathways involve modifications in drug efflux, DNA repair, escape from apoptosis, immune system evasion, improvised and differential metabolisms, drug target mutations, and epigenetic alterations [8].

Noncoding RNAs (NcRNAs) are known to regulate drug resistance in BC patients. Hence, it is critical to elucidate the correlation and underlying mechanism of the relationship governing ncRNAs and drug resistance in BC. Scientists reported that > 80% of the entire human genome undergoes transcription [9, 10]. Interestingly, only < 2% of the transcription produces functional proteins, and the rest generates ncRNAs. NcRNAs are largely separated into two categories, depending on their size and function: (1) short ncRNAs:  < 200-nucleotides long, include microRNAs (miRNAs), small interfering RNAs (siRNAs), small nucleolar RNAs (snoRNAs), and Piwi-interacting RNAs (piRNAs); and (2) long non-coding RNAs (lncRNAs): > 200-nucleotides long, transcribed via RNA polymerase II, and contains a 5’ cap, transcription start site, and polyadenylation [11]. There is a peculiar class of lncRNAs called circular RNAs (circRNAs), and they are ubiquitously found within mammals [12]. LncRNAs serve essential roles in tumor pathogenesis via both transcriptional and post-transcriptional regulation [13, 14]. In general, cytoplasmic lncRNAs modulate cell signaling, as well as transcript stability or protein translation, while nuclear lncRNAs regulate chromatin associations, as well as transcriptional and mRNA stability regulation [15]. MiRNAs belong to a category of small ncRNA that suppress protein-coding gene expression by targeting respective transcripts [16]. Several studies suggested that ncRNAs modulate gene expression at the epigenetic, transcriptional, post-transcriptional, translational and even sub-cellular localization levels [17]. Therefore, ncRNAs are known to regulate multiple facets of BC progression like cell proliferation, angiogenesis, epithelial-mesenchymal transition (EMT), cancer stem cells (CSCs), drug resistance, and metastasis [17].

In this report, we performed a review of the detailed mechanisms behind the ncRNAs-mediated regulation of chemo-, endocrine-, and targeted therapeutic resistance in BC. Moreover, our review identified possible therapeutic targets that may potentially diminish drug resistance or enhance BC treatment efficacy.

## NcRNAs regulate chemotherapeutic resistance in BC

Chemotherapy is a well-known and effective BC treatment that improves prognosis and OS of patients [18]. Chemotherapy includes anthracyclines and/or taxane administration, and in select patients, cyclophosphamide, methotrexate, and/or 5-flurouracil (5-FU) are used [19]. The mechanism underlying chemoresistance likely involves both genetic and epigenetic alterations like drug-driven mutations, drug metabolic enzyme abnormalities, cell-cycle- and apoptosis- related genes, DNA methylation, and histone modifications [20]. Moreover, most chemotherapeutic medications destroy DNA, and in response, cells elicit a DNA damage response (DDR), which may inadvertently induce drug resistance [21]. In addition, drug efflux is a commonly examined mechanism of cancer drug resistance, and enhanced drug efflux is commonly present in multidrug resistance (MDR) [22, 23]. Up-regulations in the levels of ATP-binding cassette (ABC) superfamily members like P-gp (ABCB1), multidrug-resistance-associated protein 1 (MRP1/ABCC1), multidrug-resistance-associated protein 7 (MRP7/ABCC10), and BC resistance protein (BCRP/ABCG2) are frequently observed in drug resistance associated with various forms of cancers [24–26].

### NcRNAs promote chemoresistance

**Fig. 1:**
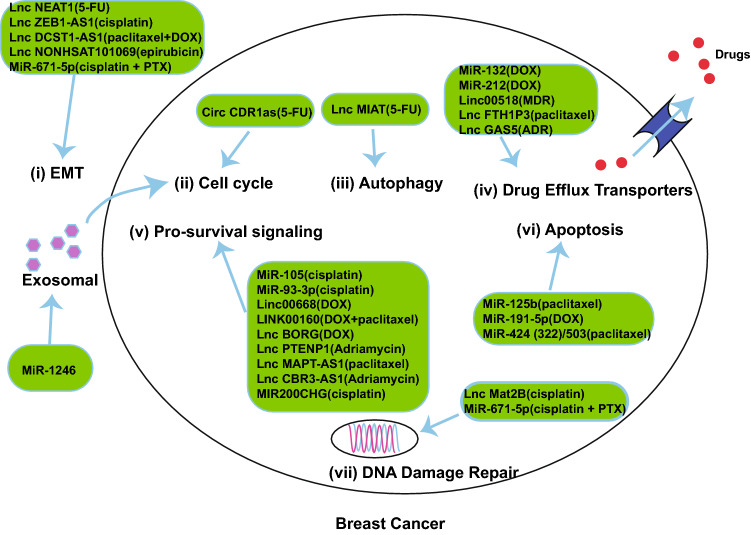
NcRNAs promote chemo-resistance

The mechanisms of ncRNAs promoting chemoresistance are summarized based on the following aspects: (i) EMT, (ii) cell cycle, (iii) autophagy, (iv) drug efflux transporters, (v) pro-survival signaling pathways, (vi) apoptosis, and (vii) DNA damage repair (Fig. 1 and Table 1).

**Table 1:** NcRNAs promote chemotherapeutic resistance in breast cancer

NcRNAs	Expression	Target gene	Drug	Refs
LINK00160	Upregulation	C/EBPβ/TEF3	Paclitaxel and DOX	[28]
LncMIAT	Upregulation	AKT	5-FU	[33]
LncNEAT1	Upregulation	Unknown	5-FU	[35]
LncRNA BORG	Upregulation	RPA1/NF-κB	Chemotherapy	[36]
LncNONHSAT101069	Upregulation	MiR-129-5p;Twist1	Epirubicin	[37]
LncRNA FTH1P3	Upregulation	MiR-206/ABCB1	Paclitaxel	[38, 39]
Linc00518	Upregulation	MiR-199a/MRP1	MDR	[42]
LncZEB1-AS1	Upregulation	MiR-129-5p/ZEB1	Cisplatin	[45]
LncPTENP1	Upregulation	MiR-20a/PTEN/PI3K/Akt	ADR	[47]
LncMat2B	Upregulation	DNA damage; ROS	Cisplatin	[48]
Linc00668	Upregulation	SND1	DOX	[53]
LncMAPT-AS1	Upregulation	MAPT	Paclitaxel	[56]
LncRNA DCST1-AS1	Upregulation	ANXA1	DOX and Paclitaxel	[57, 58]
LncRNA CBR3-AS1	Upregulation	JNK1/MEK4/MAPK	ADR	[59]
MIR200CHG	Upregulation	YB-1	Cisplatin	[60]
LncRNA GAS5	Upregulation	MiR-221-3p/DKK2; Wnt/β-catenin	ADR	[63]
CircCDR1as	Upregulation	MiR-7/CCNE1	5-FU	[65]
MiR-191-5p	Upregulation	SOX4	DOX	[66, 67]
MiR-105/93-3p	Upregulation	SFPR1/Wnt/β-catenin	Cisplatin	[68]
MiR-132/212	Upregulation	PTEN/AKT/NF-Κb/BCRP	DOX	[69]
MiR-424(322)/503	Downregulation	BCL-2;IGF1R	Paclitaxel	[70]
MiR-125b	Upregulation	Bak1	Paclitaxel	[71]
MiR-671-5p	Downregulation	FOXM1	Cisplatin and Paclitaxel	[76]
MiR-1246	Upregulation	CCNG2	Chemotherapy	[80, 81]

Either up-regulated (↑) or down-regulated (↓) in chemotherapy resistant BC cells

#### LINK00160

Abnormally expressed trefoil factor 3 (TFF3) enhances oncogenesis of prostate cancer cells [27]. In addition, LINC00160 overexpression was shown to increase TFF3 levels via C/EBPβ regulation. In doing so, MCF-7 cells were made to be resistant to paclitaxel (PTX) and BT474 cells to doxorubicin (DOX) [28].

#### LncMIAT

Autophagy is a cellular process that is induced by nutrient deprivation, endoplasmic reticulum stress (ERS), and hypoxia [29]. The ERS is intricately linked to drug resistance in BC [30–32]. 5-FU induces BC cell resistance via induction of ERS. As a result, the GRP78/OCT4/lncRNA MIAT/AKT pathway is activated [33].

#### LncNEAT1

HMGA2 is reported to regulate EMT transcription factors (TFs) in BC patients [34]. LncRNA NEAT1 promotes cell proliferation using the miR-211/HMGA2 pathway in BC patients. They also revealed that NEAT1 suppression enhances 5-FU responsiveness to BC [35].

#### LncRNA BORG

LncRNA BORG levels are very susceptible to cytotoxic medications, and promotes a transcriptional response that mediates survival and chemoresistance of TNBC cells. Mechanically, the chemo-resistant BORG traits depend on the robust activation of the NF-κB axis via a new BORG-based feedback loop, and via its ability to interact with and activate RPA1 [36].

#### LncRNA NONHSAT101069

Overexpressing lncRNA NONHSAT101069 enhances epirubicin resistance and EMT processing of BC cells. In terms of underlying mechanism, NONHSAT101069 functions as a competing endogenous RNA (ceRNA) and sequesters miR-129-5p, which, in turn, promotes epirubicin resistance, metastasis, and EMT processing of BC cells via the Twist1 axis [37].

#### LncRNA FTH1P3

FTH1P3 upregulation accelerates cell proliferation, migration, cell cycle and migration via suppression of miR-224-5p in uveal melanoma cell lines [38]. FTH1P3 levels are enriched in PTX-resistant BC tissue specimen and cells. Mechanically, FTH1P3 serves as a ceRNA and sequesters miR-206 to augment ABCB1 protein concentration [39].

#### Linc00518

MRP1 which originated from the ABCC1 gene, belongs to the ABC transporter superfamily residing on chromosome 16p13.1. Elevated MRP1 levels enhance MDR in BC [40, 41]. Linc00518 induces MDR in BC by modulating the miR-199a/MRP1 network [42].

#### LncZEB1-AS1

ZEB1-driven BC progression occurs via acceleration of EMT, tumor pathogenesis, and angiogenesis [43, 44]. LncRNA ZEB1-AS1 is ubiquitously expressed in BC. In addition, researchers demonstrated that ZEB1-AS1 deficiency drastically reduces ZEB1 content by up-regulating miR-129-5p, which, ultimately enhances drug sensitivity to cisplatin in BC [45].

#### LncPTENP1

There is evidence of considerable homology between lncRNA PTENP1 and the upstream section of the 3’untranslated region (UTR) of phosphatase and tensin homologs (PTEN). As such, lncPTENP1 readily modulates PTEN levels, which, in turn, affects cancer pathogenesis [46]. PTENP1 modulates Adriamycin (ADR) chemoresistance by interacting with miR-20a via the PTEN/PI3K/Akt network in BC [47].

#### LncMat2B

LncMat2B is ubiquitously expressed in the cisplatin-resistant MCF-7 cell line. Moreover, its incorporation into wild type MCF-7 cells reduces sensitivity to cisplatin exposure by diminishing DNA damage and reactive oxygen species (ROS) formation [48].

#### Linc00668

SND1 is crucial for tumor progression in BC [49–52]. Linc00668 promotes BC cell resistance to DOX via interaction with SND1. This enables the expression of downstream SND1 targets [53].

#### LncRNA MAPT-AS1

MAPT is strongly correlated with PTX resistance in BC [54, 55]. MAPT-AS1 is an antisense MAPT transcript, and it is co-expressed with MAPT. Mechanically, MAPT-AS1 overexpression partially protects MAPT transcripts from degradation, and vice versa. Conversely, MAPT-AS1 knock-down makes cancer cells more susceptible to PTX by modulating MAPT levels in ER-negative BC [56].

#### LncRNA DCST1-AS1

Annexin A1 (ANXA1) modulates cancer cell proliferation, apoptosis, invasion, and metastasis [57]. DCST1-AS1 induces transforming growth factor β (TGF β)-triggered EMT, and augments DOX and PTX resistance in TNBC cells using ANXA1 [58].

#### LncRNA CBR3-AS1

LncRNA CBR3 antisense RNA 1 (CBR3-AS1) induces chemotherapeutic (ADR) resistance of BC by serving as a ceRNA via the JNK1/MEK4-based mitogen-activated protein kinase (MAPK) network [59].

#### MIR200CHG

Cellular and animal models revealed that MIR200CHG induces BC cisplatin resistance. Mechanically, MIR200CHG physically interacts with the TF Y-box binding protein-1 (YB-1), and prevents its ubiquitination-mediated destruction. MIR200CHG modulates YB-1-mediated phosphorylation at serine 102, which, in turn, influences expression of tumor cell cisplatin resistance-related genes [60].

#### LncRNA GAS5

P-gp/ABCB1 overexpression increases energy-based cytotoxic drug efflux from cancer cells, thereby enhancing drug resistance [61, 62]. A recent study revealed that GAS5 restores the ABCB1-induced ADR resistance using the miR-221-3p/DKK2 pathway, and by suppressing the Wnt/β-catenin network [63].

#### CircCDR1as

CDR1as serves as a miR-7 suppressor in the developing midbrain of zebrafish [64]. Mechanically, circRNACDR1as decreases 5-FU chemo-responsiveness in BC by sequestering miR-7 to modulate CCNE1 [65].

#### MiR-191-5p

Researchers revealed that miR-191-5p is a negative apoptosis modulator in BC. In addition, SOX4 was shown to influence apoptosis in BC [66]. MiR-191-5p directly targets SOX4. Mechanically, the P53-miR-191-SOX4 axis modulates drug resistance in BC. In contrast, anti-miR-191 treatment makes BC cells more susceptible to the DOX-mediated apoptotic death [67].

#### MiR-105 and MiR-93-3p

MiR-105 and miR-93-3p are generally elevated and associated with worse outcome in TNBC. Mechanically, miR-105/93-3p promotes cisplatin resistance in BC by activating the Wnt/β-catenin network while down-regulating SFPR1 [68].

#### MiR-132 and MiR-212

MiR-132/-212 are ubiquitously expressed in DOX-resistant BC. MiR-132/-212 overexpression induces BCRP-induced DOX efflux in MCF-7 cells. Moreover, miR-132/-212 overexpression in MCF-7/ADR cells suppresses PTEN levels, while activating AKT phosphorylation and the NF-κB axis, which, in turn, augments BCRP content [69].

#### MiR-424 (322)/503

The miR-424 (322)/503 cluster contains both miR-424 (322) and miR-503. MiR-424 (322)/503 is often absent in a subcategory of aggressive BCs. MiR-424(322)/503 deficiency enhances PTX chemoresistance owing to the elevation of pro-apoptotic BCL-2 and insulin-like growth factor-1 receptor (IGF1R) [70].

#### MiR-125b

MiR-125b is commonly elevated in PTX-resistant cells. Mechanically, miR-125b promotes resistance of BC cells to PTX via inhibition of the pro-apoptotic BCL-2 antagonist killer 1 (Bak1) expression [71].

#### MiR-671-5p

Forkhead box protein M1 (FOXM1) is a TF that regulates drug resistance in BC cells by activating DNA damage repair networks [72–75]. MiR-671-5p deficiency, in contrast, activates the FOXM1-triggered EMT progression while enhancing DNA repair, and increasing chemoresistance (cisplatin and PTX) [76].

#### MiR-1246

MiR-1246 functions as an oncogene in cancer [77, 78]. Cyclin G2 (CCNG2) is modulated via the cell cycle and serves as a tumor-suppressor gene [79] and its expression is drastically diminished in BC [80]. Exosomal miR-1246 incorporation induces drug resistance by regulating CCNG2 expression in BC [81].

### NcRNAs promotes chemotherapeutic sensitivity

**Fig. 2:**
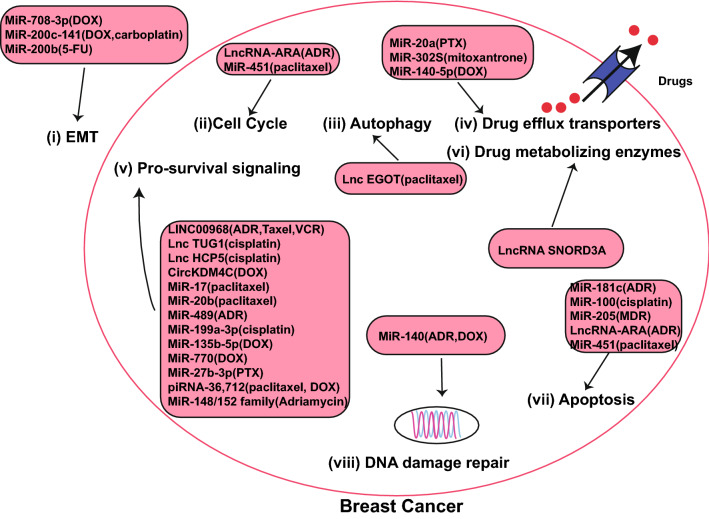
NcRNAs promote chemo-sensitivity

The mechanisms of ncRNAs promoting chemotherapeutic sensitivity using the following factors: (i) EMT, (ii) cell cycle (arrest), (iii) autophagy, (iv) drug efflux transporters, (v) pro-survival signaling pathways, (vi) drug metabolic enzymes, (vii) apoptosis, and (viii) DNA damage repair (Fig. 2 and Table 2).

**Table 2:** NcRNAs promote chemotherapeutic sensitivity in breast cancer

NcRNAs	Expression	Target gene	Medicine	Refs
LncTUG1	Upregulation	MiR-197/NLK	Cisplatin	[83]
LncRNA EGOT	Upregulation	ITPR1	Paclitaxel	[86]
LncRNA-ARA	Downregulation	Apoptosis;G2/M arrest	ADR	[87]
LINC00968	Upregulation	Wnt/β-catenin	ADR, Taxel, VCR	[89]
Lnc HCP5	Upregulation	PTEN	Cisplatin	[90]
Lnc SNORD3A	Upregulation	MiR-185-5p/UMPS	5-FU	[91]
CircKDM4C	Upregulation	MiR-548p/PBLD	Doxorubicin	[93]
MiR-17	Upregulation	NCOA3;JAB1	Paclitaxel	[95, 97]
MiR-20b	Upregulation	NCOA3	Paclitaxel	[95]
MiR-708-3p	Upregulation	CDH2, ZEB1, Vimentin	DOX	[98]
MiR-20a	Upregulation	MAPK1/P-gp/c-Myc	PTX	[99]
MiR-205	Upregulation	VEGFA/FGF2	Docetaxel, DOX, Cyclophosphamide	[101]
MiR-489	Upregulation	SPIN1/PI3K/Akt	ADR	[103]
MiR-199a-3p	Upregulation	TFAM	Cisplatin	[104]
MiR-181c	Upregulation	OPN/P53	ADR	[106]
MiR-135b-5p	Upregulation	AGR2	DOX	[108]
MiR-302S	Upregulation	BCRP	Mitoxantrone	[112]
MiR-140	Upregulation	FEN1	DOX and ADR	[117]
MiR-140-5p	Upregulation	ABCB1	DOX	[118]
MiR-200c-141	Upregulation	EMT	DOX and Carboplatin	[120]
MiR-770	Upregulation	STMN1	DOX	[123]
MiR-27b-3p	Upregulation	MAPK/Erk;PI3K/Akt	PTX	[126]
MiR-200b	Upregulation	ARRDC3	5-FU	[128]
MiR-100	Upregulation	HAX1	Cisplatin	[134]
MiR-148/152 family	Upregulation	SPIN1	ADR	[135]
MiR-451	Upregulation	YWHAZ/β-catenin	PTX	[139]
piRNA-36,712	Upregulation	SEPW1	PTX and DOX	[140]

Either up-regulated (↑) or down-regulated (↓) in chemotherapy sensitive breast cancer cells

#### LncTUG1

NLK is a negative modulator of the WNT network [82]. LncRNA TUG1 mediates its action through the regulation of the miR-197/NLK axis to enhance cisplatin sensitivity in TNBC patients [83].

#### LncRNA EGOT

Eosinophil granule ontogeny transcript (EGOT) is generated/released by ITPR1, a ligand-gated ion channel involved in the calcium secretion from the intracellular storage [84, 85]. LncRNA EGOT augments autophagy, which, in turn, makes BC more susceptible to PTX cytotoxicity, owing to an elevation in ITPR1 levels [86].

#### LncRNA-ARA

LncRNA-ARA regulates cell adhesion- and cell cycle progression-linked axes. Jiang et al*.* reported that ARA deficiency reverses drug resistance, and suppresses cell proliferation, migration, while promoting apoptosis and G2/M arrest in ADR-resistant cells [87].

#### LINC00968

WNT2 is a major Wnt ligand that regulates placental development [88]. LINC00968 reduces drug resistance (ADR, PTX and Vincristine) in BC by sequestering WNT2 by recruiting HEY1, thereby, suppressing the Wnt/β-catenin axis [89].

#### LncRNA HCP5

HCP5 was drastically reduced in MDA-MB-231/cisplatin cells, relative to the MDA-MB-231 cells. HCP5 deficiency induces cisplatin resistance in MDA-MB-231 cells by suppressing PTEN levels. Conversely, HCP5 overexpression reverses cisplatin resistance in MDA-MB-231/DDP cells by increasing PTEN levels [90].

#### LncRNA SNORD3A

Uridine monophosphate synthetase (UMPS) is a 5-FU metabolism-related gene. Mechanically, lncRNA SNORD3A sensitizes BC cells to 5-FU by sequestering miR-185-5p to augment UMPS levels [91].

#### CircKDM4C

PBLD overexpression is correlated with the suppression of multiple signal networks (Vascular endothelial growth factor A [VEGFA], MAPK, NF-κB, EMT, and angiogenesis) [92]. CircKDM4C abrogates doxorubicin resistance by modulating the miR-548p/PBLD network in BC [93].

#### MiR-17 and MiR-20b

Nuclear receptor coactivator 3 (NCO3) is a nuclear receptor coactivator which accelerates BC tumor pathogenesis by increasing the ER and PR transcriptional activities [94]. Moreover, miR-17 and miR-20b deficiencies induce PTX resistance in BC by up-regulating NCOA3 levels [95]. In addition, JAB1 is ubiquitously found in BC, and it activates pro-survival cellular networks to confer tamoxifen resistance in ERα-positive BC [96]. MiR-17 also suppresses JAB1’s oncogenic activity, which results in the suppression of tumor development while sensitizing TNBC cells to chemotherapeutic treatments [97].

#### MiR-708-3p

MiR-708-3p is an anti-cancer miRNA that is inversely associated with BC chemoresistance. MiR-708-3p restoration improves BC chemosensitivity (DOX) by inhibiting EMT via regulating CDH2, ZEB1, and vimentin (EMT stimulators) levels [98].

#### MiR-20a

MiR-20a overexpression sensitizes BC cells to chemotherapeutic medications (PTX). Mechanically, miR-20a physically interacts with the 3’ UTR of MAPK1, thereby down-regulating levels of P-gp and c-Myc by suppressing the MAPK/ERK network. In the meantime, c-Myc binds to the promoter of the miR-20a gene to induce transcription of the miR-20a gene [99].

#### MiR-205

VEGFA and fibroblast grow factor-2 (FGF2) are the strongest modulators of angiogenesis [100]. MiR-205 greatly improves chemosensitivity of BC cells to neoadjuvant chemotherapy (docetaxol, DOX, and cyclophosphamide) by diminishing both VEGFA and FGF2 levels, thereby increasing cellular apoptosis evasion [101].

#### MiR-489

SPIN1 was identified to involve in tumorigenesis [102]. MiR-489 is scarcely expressed in drug resistant BC. Mechanically, miR-489 enhances chemosensitivity (ADR) via the SPIN1/PI3K/Akt network [103].

#### MiR-199a-3p

MDA-MB-231/cisplatin exhibited a significantly lower expression level of miR-199a-3p compared with its parental cell line MDA-MB-231. MiR-199a-3p regulates mitochondrial transcription factor A (TFAM) levels. TFAM strongly regulates drug resistance (cisplatin) and tumor progression, by suppressing TFAM 3’UTR activity [104].

#### MiR-181c

Osteopontin (OPN) is excessively expressed in cancer cell lines that are prone to metastasis [105]. MiR-181c increases chemosensitivity (ADR) via diminishing OPN levels, which, in turn, enhances p53-based transactivation and apoptosis in resistant BC cells [106].

#### MiR-135b-5p

Anterior gradient 2 (AGR2) regulates BC pathogenesis, particularly, growth, drug resistance, and metastasis [107]. Mechanically, miR-135b-5p sequesters AGR2 to augment DOX-responsiveness of BC cells [108].

#### MiR-302S

BCRP eliminates its substrate anti-cancer drugs to induce MDR in cancer cells [109]. MiR-302a-d is also termed “miR-302S”, owing to the same seed sequence (5’-aagugcu-3’) [110, 111]. MiR-302S down-regulates BCRP expression to enhance chemosensitivity (mitoxantrone) of BC [112].

#### MiR-140

FEN1 regulates genomic stability and integrity via participation in multiple DNA repair pathways (BER, NHEJ, HRR and NER) [113–116]. MiR-140 suppresses FEN1 levels via direct interaction with its 3’ UTR, which results in dysfunctional DNA repair and impaired BC progression. MiR-140 overexpression makes BC cells more susceptible to chemotherapeutic drugs (DOX and ADR) targeting BC [117].

#### MiR-140-5p

Wnt1 belongs to the Wnt family, and accelerates cell cycle, migration, and survival. MiR-140-5p induces chemosensitivity to DOX in BC stem cells (BCSCs) via suppression of ABCB1 levels [118].

#### MiR-200c-141

The miR-200 family is a critical modulator of EMT [119]. In a study, miR-200c-141 cluster overexpression in an in vivo CSC-enriched claudin-low tumor model, reduced tumor development and stem cell functionality, thus resulting in the absence of EMT characteristics, along with an enhancement of chemotherapeutic (DOX and carboplatin) sensitivity [120].

#### MiR-770

Stathmin1 (STMN1) induces microtubule depolymerization by sponging tubulin and activating catastrophes [121, 122]. MiR-770 directly targets and diminishes STMN1 levels to suppress chemoresistance (DOX) in TNBC cells [123].

#### MiR-27b-3p

CBLB is an upstream factor of the PI3K/Akt network. It regulates sensitivity of cetuximab in gastric cancer [124]. GRB2, another essential upstream factor in the MAPK/Erk network is known to resist ovarian cancer therapy by cisplatin. This occurs through the activation of the MAPK/Erk network [125]. Mechanically, miR-27b-3p reverses the PTX-mediated resistance by specifically reducing its target genes (CBLB and GRB2), and thus down-regulating the MAPK/Erk and PI3K/Akt networks [126].

#### MiR-200b

Arrestin domain containing 3 (ARRDC3) is scarcely expressed in metastatic TNBC cells owing to epigenetic silencing [127]. ARRDC3 inverses EMT characteristics and chemo-resistance (5-FU) of TNBC cells by increasing miR-200b levels [128].

#### MiR-100

MiR-100 promotes cancer apoptosis [129, 130]. HAX1 (an anti-apoptotic protein) overexpression induces chemoresistance in BC [131–133], whereas, miR-100 overexpression enhances responsiveness of MDA-MB-231/R and MCF-7/R cells to cisplatin treatment, while promoting cisplatin-driven mitochondrial apoptosis by regulating HAX1 [134].

#### MiR-148/152 family

Spindlin (SPIN) is up-regulated in chemo-resistant BC tissues, and participates in the PI3K/Akt-based chemoresistance [103]. The miR-148/152 family targets SPIN1 in BC. As a result, miR-148a-3p, miR-148b-3p, and miR-152-3p enhance ADR responsiveness by modulating SPIN1 in BC [135].

#### MiR-451

β-catenin is central to the Wnt/β-catenin network. Upon activation of Wnt signaling, β-catenin is rescued from degradation, resulting in its accumulation in the cytoplasm, followed by its translocation to the nucleus, activation of target genes (c-Myc and cyclin D1), which ultimately enhances tumor pathogenesis [136–138]. MiR-451 accelerates apoptosis and cell-cycle arrest of PTX-resistant cells via direct binding of the YWHAZ/β-catenin network [139].

#### PiRNA-36,712

PiRNA-36,712 restrains BC chemoresistance. Mechanically, piRNA-36,712 binds to SEPW1P transcript, thereby decreasing SEPW1 expression via sponging by miR-7 and miR-324. In addition, piRNA-36,712 elicits a combined anticancer effect with PTX and DOX [140].

## NcRNAs with endocrine therapy resistance in BC

Approximately 70% of all BC patients exhibit ubiquitous ER expression [141, 142]. As such, it is a promising target for endocrine therapy. Two major ER isoforms (ERα and ERβ), encoded by 2 distinct genes (ESR1 and ESR2), regulate the nuclear and extranuclear ER axes [143, 144]. At present, three forms of endocrine therapies are used in clinics: (a) aromatase inhibitors (AI), (b) selective ER modulators (SERMs) and (c) selective ER degraders (SERDs) that antagonize ER [145]. The first of these SERMs is tamoxifen, a drug used frequently till this day to treat ER-positive patients. However, patients soon become resistant to this drug, which limits its use [146, 147]. AI blocks the enzyme aromatase, which regulates estrogen production. This prevents the development of hormone-receptor-positive BC cells. AI is primarily employed in postmenopausal women, and it performs better than tamoxifen in this demographic [148]. Fulvestrant is the preferred SERD for treating cancer patients. Both preclinical and clinical trials revealed that this is effective even in the tamoxifen-resistant (TR) models, and do not elicit agonistic activity in oestrogen-sensitive tissues like the endometrium [149, 150]. Scientists uncovered several underlying mechanisms that produce endocrine resistance, namely, deregulation of the classical estrogen signaling, activation of growth factor receptor networks, changes in the cell cycle and apoptotic process, and epigenetic modification [151].

**Fig. 3:**
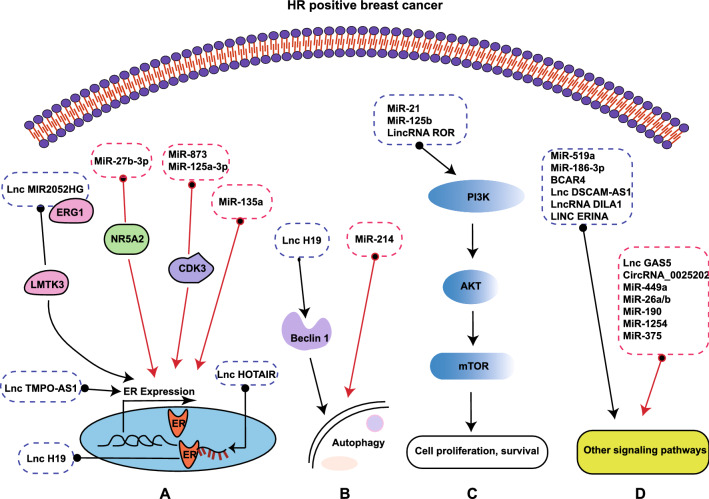
NcRNAs regulate response to endocrine therapy

Herein, we detailed the ncRNAs-related pathways involved in endocrine therapy resistance and sensitivity, particularly, in terms of the dysregulated signaling pathways: (i) ER signaling pathway, (ii) autophagy signaling pathway, (iii) PI3K/Akt/mTOR signaling pathway, (iv) and other pro-survival signaling pathways (Fig. 3 and Table 3).

**Table 3:** NcRNAs promote resistance or sensitivity to endocrine therapy in breast cancer

NcRNAs	Expression	Target signaling pathway	Medicine	Refs
LncMIR2052HG	Upregulation	ERG1/LMTK3/PKC/MEK/ERK/RSK1; PKC/AKT/FOXO3 ERα	AI (Resistance)	[152]
LncRNA HOTAIR	Upregulation	ER-target genes	Tamoxifen (Resistance)	[153]
BCAR4	Upregulation	HER2	Tamoxifen (Resistance)	[154]
LncRNA H19	Upregulation	SAHH/DNMT3B/Beclin; ERα	Tamoxifen, Fulvestrant (Resistance)	[156, 157]
LincRNA-ROR	Upregulation	PI3K/Akt/mTOR	Tamoxifen (Resistance)	[159]
LncRNA DSCAM‐AS1	Upregulation	MiR-137/EPS8	Tamoxifen (Resistance)	[161]
LncRNA TMPO-AS1	Upregulation	ESR1	Endocrine therapy (Resistance)	[162]
LncDILA1	Upregulation	Cyclin D1 phosphorylation	Tamoxifen (Resistance)	[163]
LINC ERINA	Upregulation	E2F1	CDK inhibitors (Resistance)	[164]
MiR-125b	Upregulation	AKT/mTOR	Letrozole (Resistance)	[168]
MiR-519a	Upregulation	PTEN, RB1, CDKN1A/p21	Tamoxifen (Resistance)	[169]
MiR-186-3p	Upregulation	EREG	Tamoxifen (Resistance)	[173]
MiR-21	Upregulation	PI3K/Akt/mTOR	Tamoxifen, Fulvestrant (Resistance)	[175]
LncRNA GAS5	Downregulation	MiR-222/GAS5/PTEN	Tamoxifen (Sensitivity)	[177]
CircRNA_0025202	Upregulation	MiR-182-5p/FOXO3a	Tamoxifen (Sensitivity)	[179]
MiR-449a	Upregulation	ADAM22	Tamoxifen (Sensitivity)	[181]
MiR-27b-3p	Upregulation	NR5A2, CREB1	Tamoxifen (Sensitivity)	[185]
MiR-873	Upregulation	CDK3	Tamoxifen (Sensitivity)	[186]
MiR-125a-3p	Upregulation	CDK3	Tamoxifen (Sensitivity)	[188]
MiR-26a/b	Upregulation	ERBB2	Tamoxifen (Sensitivity)	[190]
MiR-190	Upregulation	SOX9	Endocrine therapy (Sensitivity)	[191]
MiR-214	Upregulation	UCP2	Tamoxifen, Fulvestrant (Sensitivity)	[194]
MiR-1254	Upregulation	CCAR1	Tamoxifen (Sensitivity)	[197]
MiR-135a	Upregulation	ERα	Tamoxifen (Sensitivity)	[200]
MiR-375	Upregulation	MTDH	Tamoxifen (Sensitivity)	[199]

### NcRNAs promotes endocrine therapy resistance

#### LncMIR2052HG

MIR2052HG directly interacts with the early growth response protein 1 (ERG1) protein to increase LMTK3 expression, thereby sustaining ESR1 levels and stabilized ERα protein, thus leading to AI resistance. Mechanistically, LMTK3 regulates ERα stability via the PKC/MEK/ERK/RSK1 pathway and ERα expression via the PKC/AKT/FOXO3 network [152].

#### LncRNA HOTAIR

HOTAIR is markedly elevated in tumors of TR BC patients, relative to their primary tumors prior to treatment. Direct association between HOTAIR and ER results in high levels of nuclear ER, even under estrogen-depleted conditions. This enables ER genomic targeting and induces transcription of the ER-target genes. Hence, HOTAIR augments the ER axis, and elicits tamoxifen resistance in BC [153].

#### BCAR4

BCAR4 accelerates BC progression. Godinho et al*.* reported that BCAR4 levels in BC are strongly correlated with aggressiveness and tamoxifen resistance via regulation of the HER2 axis [154].

#### LncRNA H19

Autophagy is a potential mechanism for tamoxifen resistance. Beclin1 (a key mediator of autophagy) overexpression makes cells unresponsive to estrogen-based signaling, which leads to tamoxifen resistance in BCs [155]. H19 overexpression augments autophagy and induces tamoxifen resistance in ER-positive BC cells by diminishing methylation in the Beclin 1 promotor region using the H19/SAHH/DNMT3B network [156]. In addition, H19 deficiency makes endocrine therapy resistant (ETR) cells susceptible to tamoxifen and fulvestrant, in an H19-dependent manner. H19 also modulates ERα levels in ETR cells, and protects against fulvestrant-based apoptosis [157].

#### LincRNA-ROR

LincRNA-ROR regulates BC metastasis [158]. LincRNA-ROR deficiency enhances MDA-MB-231 cell sensitivity to tamoxifen by inhibiting PI3K/Akt/mTOR activity [159].

#### LncRNA DSCAM‐AS1

Epidermal growth factor receptor pathway substrate 8 (EPS8) modulates cancer cell proliferation and apoptosis [160]. DSCAM‐AS1 induces tamoxifen resistance in BC, and is inversely proportional to miR-137 levels, and directly proportional to EPS8 levels in tamoxifen-resistant BC [161].

#### LncRNA TMPO-AS1

TMPO-AS1 is ubiquitously expressed in ER-positive BCs from tamoxifen-treated patients. Mechanically, TMPO-AS1 augments the estrogen axis by stabilizing the ESR1 transcript, encoding ERα, and via direct RNA: RNA association with the 3’UTR of ESR1 [162].

#### DILA1

DILA1 binds to Cyclin D1, and is ubiquitously expressed in tamoxifen-resistant BC. Mechanistically, DILA1 prevents Cyclin D1 phosphorylation at Thr286 via direct association with Thr286, which blocks its degradation, thus enhancing Cyclin D1 levels in BC [163].

#### LINC ERINA

High lincRNA ERINA levels are strongly associated with worse ER-positive BC patient outcome and responsiveness to CDK inhibitors in BC cell lines. Mechanistically, ERINA is induced by estrogen, and promotes cell cycle progression by regulating the TF E2F1 [164].

#### MiR-125b

The AKT/mTOR axis regulates AI resistance [165–167]. Silencing miR-125b in letrozole-resistant cells prevents the constitutive activation of the AKT/mTOR axis, and overcomes letrozole resistance, by sensitizing cells to the AI treatment [168].

#### MiR-519a

PTEN, CDKN1/p21, and retinoblastoma protein (RB1) are directly targeted by miR-519a. Mechanically, tamoxifen-resistant cells express high levels of miR-519a, which blocks the expressions of PTEN, RB1, and CDKN1A/p21, thus enabling cells to proliferate, even after tamoxifen exposure [169].

#### MiR-186-3p

EGFR signaling is also crucial for developing tamoxifen resistance in BC cells [170, 171]. Epiregulin (EREG) induces EGFR homodimerization, which initiates downstream signaling to promote cell proliferation [172]. MiR-186-3p targets EREG in BC. Moreover, the miR-186-3p/EREG network produces tamoxifen resistance and aerobic glycolysis in ER-positive BC [173].

#### MiR-21

Aberrant expression of miR-21 involved in chemoresistance of tumor [174]. Silencing of miR-21 confers the sensitivity to tamoxifen and fulvestrant by enhancing autophagic cell death through inhibition of the PI3K/AKT/mTOR by targeting PTEN [175].

### NcRNAs promotes endocrine therapy sensitivity

#### LncRNA GAS5

PTEN regulates tamoxifen responsiveness in BC [176]. MiR-222 sequesters GAS5, suppresses PTEN, and enhances BC sensitivity to tamoxifen [177].

#### CircRNA_0025202

FOXO3a was downregulated in BC [178]. CircRNA_0025202 was significantly downregulated in MCF-7/TR cells. In terms of mechanism, circRNA_0025202 promotes tamoxifen sensitization via miR-182-5p/FOXO3a axis [179].

#### MiR-449a

A disintegrin and metalloproteinase (ADAM22) promotes ER-positive BC progression [180]. Downregulation of miR-449a promotes ADAM22 expression, which induces tamoxifen resistance in BC cells [181].

#### MiR-27b-3p

Nuclear receptor subfamily 5 group A member 2 (NR5A2) enhances BC cell proliferation by interacting with the ERα promoter to initiate its expression [182]. cAMP-response element binding protein 1 (CREB1) activates essential factors related to the anti-apoptosis pathway [183, 184]. MiR-27b-3p inhibits NR5A2 and CREB1 expressions. As a result, tamoxifen-induced cytotoxicity is enhanced in BC [185].

#### MiR-873

Cyclin-dependent kinase 3 (CDK3) phosphorylated ER and enhances ER activity. MiR-873 inhibits ERα transcriptional activity and tamoxifen resistance via targeting CDK3 in BC [186].

#### MiR-125a-3p

CDK3 is a potential target of miR-125a-3p in ER-positive BC [187]. MiR-125a-3p can function as a novel tumor suppressor in ER-positive BC by targeting CDK3, which may be a potential therapeutic approach for tamoxifen resistant BC therapy [188].

#### MiR-26a/b

Hu-antigen R (HuR) is an RNA-interacting protein (RBP) which binds to the AU-rich regions in the 3’UTR of transcripts to enhance their stability [189]. Reduced miR-26a/b and enhanced HuR levels post-transcriptionally augments ERBB2 expression, which, in turn, mediates the acquired tamoxifen resistance in ER-positive BC cells [190].

#### MiR-190

MiR-190 suppresses the Wnt/β-catenin axis to enhance anti-estrogen responsiveness by regulating SRY-related high mobility group box 9 (SOX9). In addition, recent evidences suggest a mechanism involving ZEB1-miR-190-SOX9 that mediates resistance to endocrine therapy in BC. ZEB1 interacts with the miR-190 promoter region to competitively inhibit ERα interaction, which enhances resistance to endocrine therapy [191].

#### MiR-214

Overexpression of UCP2 conferred drug resistance to chemotherapy and a higher survival through downregulation of ROS [192, 193]. MiR-214 increases the sensitivity of BC cells to tamoxifen and fulvestrant through inhibition of autophagy by targeting UCP2 [194].

#### MiR-1254

Cell cycle and apoptosis regulator 1 (CCAR1) is an apoptosis mediator or transcriptional coactivator for nuclear receptors or P53. As such, it has multiple roles in regulating cancer cell progression [195, 196]. CCAR1 5’ UTR is a natural miRancer of the endogenous miR-1254, and it makes TR BC cells susceptible to tamoxifen [197].

#### MiR-135a

MiR-135a was downregulated in BC/TR [198, 199]. The decreased expression of miR-135a resulted in an increased level of the miR-135a target genes (ESR1, ESRRA, NCOA1, PIM2, MRAS, and LCP1), which we have demonstrated to be key mediators of ERK1/2 and AKT1 activation, and subsequent increased ERα transcriptional activity to promote tamoxifen resistance [200].

#### MiR-375

Metadherin (MTDH) has been involved in BC metastasis. MTDH overexpression could induce EMT and modulate invasion as well as metastasis in BC [201]. Re-expression of miRNA-375 reverses both tamoxifen resistance and accompanying EMT-like properties by targeting MTDH in BC [199].

## NcRNAs with targeted therapy resistance in BC

**Fig. 4:**
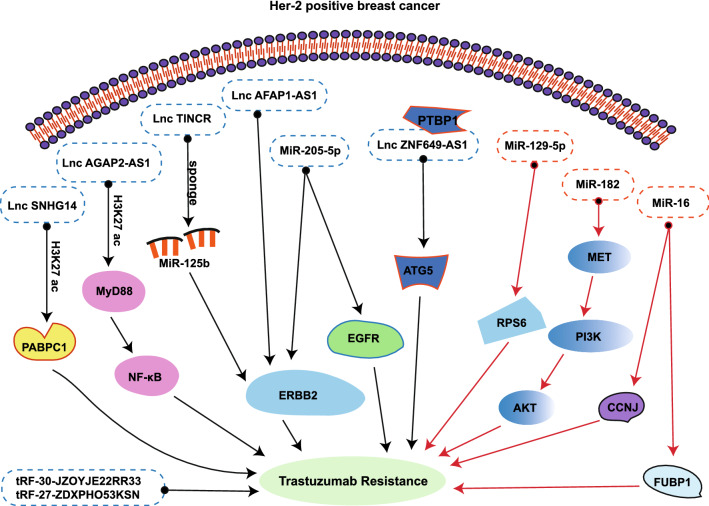
NcRNAs regulate response to targeted therapy

Erb-2/Her-2 is up-regulated in 20–30% of human invasive BCs, and is correlated with a worse patient outcome [202, 203]. In terms of monoclonal antibodies, small molecular inhibitors are used to specifically bind a target molecule. At the present time, trastuzumab, lapatinib, and pertuzumab are commonly employed for HER-2-positive BCs treatment [204]. Trastuzumab is a humanized monoclonal antibody that interacts with the HER2 receptor to suppress HER2 dimer formation, thus interrupting downstream networks, which, in turn, inhibits cell proliferation and apoptosis [148]. Lapatinib is a HER2 kinase inhibitor, which improves prognosis of HER2-amplified BC [205]. Multiple mechanisms produce resistance to targeted therapies. These include, ErbB2 levels, enhanced pro-survival signaling via alternation in tyrosine kinases receptors or intracellular signaling, which markedly enhances cell proliferation [206, 207]. Herein, we detailed the ncRNAs-mediated mechanism governing targeted therapy resistance and BC sensitivity (Fig. 4 and Table 4).

**Table 4:** NcRNAs promote resistance or sensitivity to targeted therapy in breast cancer

NcRNAs	Expression	Target gene/Signaling pathway	Medicine	Refs
LncSNHG14	Upregulation	PABPC1	Trastuzumab (Resistance)	[210]
LncAGAP2-AS1	Upregulation	MyD88/NF-κB	Trastuzumab (Resistance)	[211]
Lnc TINCR	Upregulation	MiR-125b/HER-2	Trastuzumab (Resistance)	[212]
LncRNA ZNF649-AS1	Upregulation	ATG5	Trastuzumab (Resistance)	[213]
LncRNA AFAP1-AS1	Upregulation	AUF1/ ERBB2	Trastuzumab (Resistance)	[214]
MiR-205-5p	Upregulation	p63/EGFR	Lapatinib (Resistance)	[215]
tRF-30-JZOYJE22RR33 tRF-27-ZDXPHO53KSN	Upregulation	Unknown	Trastuzumab (Resistance)	[218]
MiR-129-5p	Upregulation	rpS6	Trastuzumab (Sensitivity)	[221]
MiR-182	Upregulation	MET/PI3K/AKT/mTOR	Trastuzumab (Sensitivity)	[223]
MiR-16	Upregulation	CCNJ;FUBP1	Trastuzumab and Lapatinib (Sensitivity)	[226]

### NcRNAs promotes targeted therapeutic resistance

#### LncSNHG14

Polyadenylate‐binding proteins (PABPs) are special proteins that associate in a sequence-specific fashion with single-stranded poly (A) by RNA recognition motif (RPM). PABPC1 regulates mRNA translation and degradation [208, 209], and facilitates the stability of the 5’ cap of transcripts. Mechanically, SNHG14 induces BC trastuzumab resistance by modulating PABPC1 levels via H3K27 acetylation [210].

#### LncAGAP2-AS1

AGAP2-AS1 induces trastuzumab resistance of BC via epigenetic modulation of MyD88. Mechanically, AGAP2-AS1 interacts with the CREB-interacting protein to increase H3K27ac levels at the MyD88 promoter region, thereby up-regulating MyD88. Hence, the NF-κB axis is activated by MyD88 and AGAP2-AS1 [211].

#### Lnc TINCR

TINCR deficiency reverses trastuzumab resistance, and acquired EMT in BC. Mechanically, TINCR remains in the cytoplasm of BC cells and is sequestered by miR-125b. This, in turn, releases HER-2 and induces trastuzumab resistance [212].

#### LncRNA ZNF649-AS1

Trastuzumab treatment enhances H3K27ac levels at the ZNF649-AS1 promoter region, which elevates ZNF649-AS1, which, in turn, enhances ATG5 levels by associating with polypyrimidine tract binding protein 1 (PTBP1) to initiate its transcription. Subsequently, enhanced autophagy related 5 (ATG5) expression induces autophagy and trastuzumab resistance [213].

#### LncRNA AFAP1-AS1

AFAP1-AS1 is ubiquitously expressed in trastuzumab-resistant cells, relative to sensitive cells. Enhanced AFAP1-AS1 expression is associated with worse response and reduced survival of BC patients. Exosome-mediated AFAP1-AS1 induces trastuzumab resistance via interaction with AUF1 and activation of ERBB2 translation [214].

#### MiR-205-5p

MiR-205-5p is up-regulated in BCSCs, and directly diminishes ERBB2 expression, while indirectly reducing EGFR expression to induce to resistance to lapatinib. In addition, miR-205-5p also modulates p63 expression, which, in turn, modulates the miR-205/p63/EGFR axis [215].

#### TRF-30-JZOYJE22RR33/tRF-27-ZDXPHO53KSN

tRNA derived small RNA fragments (TRFs) regulate human cancers [216, 217]. TRF-30-JZOYJE22RR33 and TRF-27-ZDXPHO53KSN are strongly expressed in trastuzumab-resistant versus -sensitive patients, and ROC analysis revealed a strong correlation with trastuzumab resistance [218].

### NcRNAs promotes targeted therapy sensitivity

#### MiR-129-5p

Dysregulated PI3K/Akt/mTOR/rpS6 axis and PTEN deficiency contributes to trastuzumab resistance in BC [219, 220]. MiR-129-5p makes Her-2-positive BC more susceptible to trastuzumab by reducing rpS6 activity [221].

#### MiR-182

The PI3K/AKT/mTOR axis is an signaling target of MET, and it modulates multiple physiological processes [222]. MiR-182 overexpression reduces trastuzumab resistance in trastuzumab-resistant cells in part by suppressing the MET/PI3K/AKT/mTOR axis [223].

#### MiR-16

FUBP1 is a TF and RBP that modulates both transcription and translation of multiple genes [224]. CCNJ is not well characterized in mammals, and it may modulate BC [225]. MiR-16 serves as a tumor suppressor to mediate trastuzumab and lapatinib anti-proliferative effects, and CCNJ and FUBP1 are newly confirmed targets of miR-16 [226].

## Targeting oncogenic-NcRNAs to conquer drug resistance

**Fig. 5:**
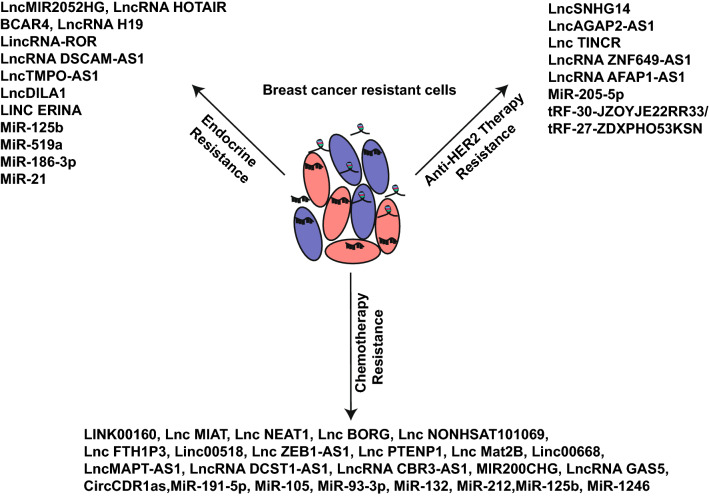
Oncogenic ncRNAs regulate drug resistance in breast cancer

In terms of the aforementioned ncRNAs-mediated drug resistance, multiple ncRNAs also possess great therapeutic target potential in future drug developments. Therefore, several researchers targeted oncogenic ncRNAs to address cancer drug resistance. Herein, we detailed the ncRNAs that are highly expressed in cancer cells, where they serve an oncogenic function to induce BC resistance to anti-cancer therapies (Fig. 5). With advancements in nanotechnology, multiple clinical trials either examined or are examining RNA-guided precision machines [227–229]. Among the annotated ncRNAs, miRNAs are most commonly examined. Additionally, lncRNAs and circRNAs were also identified as novel targets [230–232]. Double-stranded RNA-mediated interference (RNAi) and single-stranded antisense oligonucleotides (ASOs) are two main strategies that target lncRNAs. Till now, three approaches were proposed for targeting ncRNAs: ASOs, locked nucleic acids (LNAs), and morpholinos [233]. Fortunately, a clinical trial (NCT02950207) was launched to testify whether miR-100 silencing impacts patients’ response rate to hormonal treatment in BC (https://clinicaltrials.gov). Moreover, the researchers also examined miR-10b, and revealed that miR-10b LNAs enhances BC sensitivity to doxorubicin in mouse models, with no further damage to normal tissue. This suggests that reduced toxicity is strongly related to the delivery of this LNA nanoparticle [234].

## Conclusions

BC is the most common cancer among women, and the major contributor to cancer-related deaths in women [235]. Technological enhancements in early diagnosis and therapy have markedly reduced BC-related mortality, while improving patient outcome to a certain extent [236]. However, close to 35% of BC patients experience recurrence and metastasis. Moreover, they also experience resistance to chemo-, endocrine-, and radiotherapies [237, 238]. The often encountered drug resistance within BC patients severely restricts therapeutic efficacy, and negatively impacts BC patient prognosis [239]. Emerging evidences revealed that ncRNAs can function as diagnostic indicators for multiple diseases, estimator of drug response, and as targets of new drug development [240].

Herein, we summarized the dysregulated ncRNAs governing drug resistance in BC, thereby providing a comprehensive ncRNAs landscape for drug resistance in BC. Some ncRNAs regulate drug resistance and sensitivity via a complex regulatory network. For instance, lncRNA H19 modulate endocrine resistance by regulating autophagy and ERα in BC. Meanwhile, different ncRNAs also influence drug efficacy by targeting the same target molecule. For instance, PTEN modulates drug resistance by simultaneously regulating lncPTENP1, miR-132, miR-212, lncHCP5, miR-519a, GAS5, and miR-129-5p levels in BC. Several studies demonstrated a concrete mechanism of ncRNAs modulating drug resistance, however, some reports only suggested a role of few ncRNAs in regulating drug resistance. This review highlights the direction of future anti-cancer drug development, particularly, approaches that weaken drug resistance by inhibiting drug resistance-related oncogenic ncRNAs. Other studies demonstrated that ncRNAs possess great potential in treating tumor. For example, small molecules were recently shown to abrogate HOTAIR activity by interrupting the HOTAIR/EZH2 scaffold association. This offers a novel approach of inhibition with enhanced applicability in humans. EZH2 inhibitor compounds like DZNep was previously suggested as potential medications targeting solid tumors in clinics [241]. Dysregulated ncRNAs are widely present in tumor drug resistance. A clinical trial must also be launched to enhance drug sensitivity by targeting ncRNAs, as mentioned above. Hence, given the significance of ncRNAs in drug resistance, additional investigations are warranted to identify potential therapeutic targets and approaches that enhance drug sensitivity in BC.

In conclusion, we recommend an extensive investigation, involving clinical trials, to examine the mechanisms behind drug resistance, and subsequently, develop ncRNAs-based therapies to fight BC. Additionally, miRNA, circRNA and TRFs, and other ncRNAs were not reported to modulate drug resistance. However, additional investigations are needed to confirm their association, if any, with drug resistance.

## Data Availability

Not applicable.

## Abbreviations

ABC: ATP-binding cassette
ADAM: A disintegrin and metalloproteinase
ADR: Adriamycin
AI: Aromatase inhibitors
Ai-lncRNA: Antisense intronic long noncoding RNA
AGR2: Anterior gradient 2
ANXA1: Annexin A1
ARRDC3: Arrestin domain containing 3
ATG5: Autophagy related 5
Bak1: BCL-2 antagonist killer 1
BCRP: Breast cancer resistance protein
BCSCs: Breast cancer stem cells
CCAR1: Cell cycle and apoptosis regulator 1
CCNG2: Cyclin G2
CDK3: Cyclin-dependent kinase 3
ceRNA: Competing endogenous RNA
circRNA: Circular RNAs
CREB1: CAMP-response element binding protein 1
CSCs: Cancer stem cells
DDR: DNA damage response
DOX: Doxorubicin
EGOT: Eosinophil granule ontogeny transcript
EMT: Epithelial-mesenchymal transition
EPS8: Epidermal growth factor receptor pathway substrate 8
ER: Estrogen receptor
EREG: Epiregulin
ERG1: Early growth response protein 1
ETR: Endocrine therapy resistance
FGF2: Fibroblast grow factor-2
FOXM1: Forkhead box protein M1
HER2: Human epidermal growth factor receptor 2
H3K27ac: H3K27 acetylation
HuR: Hu-antigen R
IGF1R: Insulin-like growth factor-1 receptor
LA: Luminal A
LB: Luminal B
lncRNA: Long non-coding RNA
MAPK: Mitogen-activated protein kinase
MDR: Multidrug resistance
miRNA: MicroRNA
MRP: Multidrug-resistance-associated protein
MTDH: Metadherin
NBL: Normal breast-like
NCO3: Nuclear receptor coactivator 3
NR5A2: Nuclear receptor subfamily 5 group A member 2
OPN: Osteopontin
OS: Overall survival
PABPs: Polyadenylate‐binding proteins
piRNAs: Piwi-interacting RNAs
PR: Progesterone receptor
PTBP1: Polypyrimidine tract binding protein 1
PTEN: Phosphatase and tensin homologs
RB1: Retinoblastoma protein
RBP: RNA-binding protein
ROS: Reactive oxygen species
RPM: RNA recognition motif
SERDs: Selective estrogen receptor degraders
SERMs: Selective estrogen receptor modulators
siRNAs: Small interfering RNAs
snoRNA: Small nucleolar RNAs
SOX9: SRY-related high mobility group box 9
SPIN: Spindlin
STMN1: Stathmin1
TF: Transcription factor
TFAM: Mitochondrial transcription factor A
TFF3: Trefoil factor 3
TGF-β: Transforming growth factor β
TNBC: Triple negative breast cancer
TR: Tamoxifen-resistant
TRFs: TRNA derived small RNA fragments
UTR: Untranslated region
UMPS: Uridine monophosphate synthetase
VEGFA: Vascular endothelial growth factor A
ZEB1: Zinc-finger E-box binding homeobox 1
